# Emotion dysregulation and impulsivity as overlapping symptoms in adult Attention-Deficit/Hyperactivity Disorder and Borderline Personality Disorder: severity profiles and associations with childhood traumatization and personality functioning

**DOI:** 10.1186/s12991-024-00540-y

**Published:** 2025-01-13

**Authors:** Eszter Kenézlői, Lívia Balogh, Szilvia Somogyi, Evelyn E. Lévay, Zsuzsa Halmai, Zsófia Nemoda, Zsolt S. Unoka, János M. Réthelyi

**Affiliations:** 1 Doctoral School of Mental Health Sciences, Semmelweis University, 1085 Üllői út 26, Budapest, Hungary; 2 Department of Psychiatry and Psychotherapy, Semmelweis University, 1083 Balassa utca 6, Budapest, Hungary; 3 Bhaktivedanta College, 1039 Attila utca 8, Budapest, Hungary; 4 Department of Molecular Biology, Semmelweis University, 1094 Tűzoltó utca 34-47, Budapest, Hungary

**Keywords:** Adult attention-deficit hyperactivity disorder (aADHD), Borderline personality disorder (BPD), Impulsivity, Emotion dysregulation, Inhibitory control, Childhood traumatization, Level of personality functioning

## Abstract

**Background:**

Increased levels of emotion dysregulation and impulsive behavior are overlapping symptoms in adult Attention-Deficit/Hyperactivity Disorder (aADHD) and Borderline Personality Disorder (BPD), both symptom domains reflecting on inhibitory control, although from different angles. Our aims were to describe their differences in the above conditions, investigate their associations with childhood traumatization, and to explore the potential mediation of emotion dysregulation and impulsivity between childhood traumas and personality functioning.

**Methods:**

Young adults between 18 and 36 years diagnosed with aADHD (*n* = 100) and BPD (*n* = 63) were investigated with structured clinical interviews, while age-matched healthy controls (*n* = 100) were screened for psychiatric disorders. Patients with aADHD-BPD comorbidity were excluded from further analyses. The Difficulties in Emotion Regulation Scale, the Barratt Impulsiveness Scale, the Level of Personality Functioning Scale, and the Childhood Trauma Questionnaire-Short Form were administered to investigate trait measures and childhood traumatization, respectively. Behavioral impulsivity and delay aversion were assessed using selected tests of the Cambridge Neuropsychological Test Automated Battery, and a computerized decision-making paradigm based on the Rogers decision-making task, respectively.

**Results:**

Significantly higher levels of emotion dysregulation and impulsivity were present both in the aADHD and BPD groups, however with different profiles. Waiting and stopping impulsivity was selectively higher among aADHD patients compared to healthy controls. The BPD group reported higher levels of emotion dysregulation in all domains, and demonstrated increased delay aversion among uncertain conditions in decision-making. Higher levels of childhood trauma were associated with emotion dysregulation, trait impulsivity, and delay aversion across groups. Emotion regulatory capacity played a significant mediating role between childhood traumatization and the level of personality functioning.

**Conclusions:**

Inhibitory control profiles of the aADHD and BPD groups were divergent. Childhood traumatization was associated with lower levels of personality functioning in adulthood, independently of diagnosis, an effect mediated more by emotion dysregulation, rather than impulsivity. These findings have various clinical implications for the treatment of aADHD and BPD, including psychoeducation, pharmacological interventions, and psychotherapy targeting specific symptom domains.

**Supplementary Information:**

The online version contains supplementary material available at 10.1186/s12991-024-00540-y.

## Background

Elevated levels of emotion dysregulation (ED) and impulsivity are important symptoms and diagnostic criteria of Borderline Personality Disorder (BPD). Impulsive behavior constitutes a core feature in Attention Deficit Hyperactivity Disorder (ADHD) as well, while ED is only recognized as an associated symptom in the Diagnostic and Statistical Manual of Mental Disorders Fifth Edition Text Revision (DSM-5-TR) [1]. Both disorders are prevalent psychiatric conditions resulting in high levels of personal suffering and health-care burden [2].

Impulse control difficulties are also characteristic of disorders that are classified under the heading of Disruptive, Impulse-Control and Conduct Disorders in the DSM-5-TR, such as intermittent explosive disorder, kleptomania, and pyromania. They are important in several other disorders as well, e.g. bulimia, substance use disorders, behavioral addictions, non-suicidal self-harm, paraphilias and tic disorders [1]. Moreover, impulse dyscontrol constitutes a significant risk factor for suicidal behavior [3]. The most widely used definition of impulsivity decribes the symptom as a predisposition to rapid and unplanned reactions triggered by internal or external stimuli, regardless of their negative consequences for the individual or others. Computational modeling and empirical evidence demonstrated that impulsivity was closely linked to value-free random exploration, the tendency to forego highly valued and known outcomes for unknown choice options [4]. ED is considered as difficulties with or the inability to modulate any aspects of the emotion regulation process, including emotion escalation and de-escalation, to an extent that interferes with development or functioning [5].

Previous studies indicate that there is no global impulsiveness factor, rather, impulsivity is a multidimensional construct, best captured by at least three different domains [6]. The impulsivity profile consists of (1) impulsive personality traits reflecting self-reported attributions of self-regulatory capacity, (2) impulsive actions, i.e. the inability of withholding premature actions (waiting impulsivity) or stopping ongoing actions (stopping impulsivity), and (3) impulsive choice patterns, the influence of delay aversion in decision making. Impulsive choice refers to a tendency to prefer smaller, immediate rewards over larger, delayed rewards [7].

Several lines of evidence demonstrate altered self-regulatory profiles in aADHD and BPD. For example, BPD patients scored significantly higher on the Barratt Impulsiveness Scale than healthy control individuals, while the aADHD group scored significantly higher than the BPD and control groups [8]. In terms of behavioral measures of impulsivity, one small-scale study indicated increased impulsive choice in the BPD group, while increased impulsive action and cognitive deficits were observed in the aADHD group [9]. Behavioral impulsivity was clearly the most severe among patients with aADHD, and less severe among BPD patients without comorbid ADHD [10]. Delay aversion was affected both in ADHD and BPD. Current evidence suggests that “cold”, i.e., emotionally neutral impulse control is less affected in BPD than “hot” impulse control, i.e., situations involving affective and/or motivational aspects. Individuals with BPD diagnosis may be more prone to make decisions providing immediate gratification, but negative consequences in the long term.

Despite the clinical evidence of transdiagnostic similarities, to date, only a few studies have directly compared ED in ADHD and BPD. Witt et al. compared the two patient groups with a healthy control group, and found significantly higher levels of ED in BPD patients in comparison with aADHD patients [11]. Similarly, Philipsen et al. compared aADHD, BPD, and healthy control groups using the Borderline Symptom List, and showed that patients with aADHD had higher scores than controls on all 7 subscales, including Emotion Regulation, but presented lower scores than BPD participants [12]. Cavelti et al. used the Emotion Regulation Skills Questionnaire in the three subgroups, and reported comparable ED difficulties in the aADHD and BPD groups [13]. In the most recent study, aADHD patients had higher ED than people from the general population, but they demonstrated better control over their emotions with higher use of adaptive cognitive strategies and less use of non-adaptive strategies than BPD patients [14]. Research on impulsivity in aADHD and BPD is ongoing, and it remains unclear whether ED and impulsivity manifests itself differently in these conditions. Further studies and analyses are warranted to provide a clearer understanding of these relationships [15].

Several studies reflected on the role of childhood traumatization in the background of ED and impulsivity in adulthood. Krause-Utz et al. investigated associations of childhood trauma severity with emotion regulation difficulties and impulsivity in women with BPD or aADHD with comorbid substance use disorder. Childhood traumas, particularly emotional maltreatment was positively associated with ED across all groups [16]. According to the results of Lepouriel et al., trait impulsivity was significantly associated with childhood traumatization in the healthy control group, but not in the aADHD and BPD groups [7]. ED and impulsivity symptoms in children, such as motor restlessness, emotional instability, and concentration problems can also be directly linked to traumatization. According to this concept, the daydreaming behaviour often seen in children with ADHD may actually be attributable to dissociation or subconscious avoidance of trauma triggers [17].

From a clinical point of view, the severity of personality dysfunction is seen as an important measure, informative about long-term prognosis regardless of the primary diagnoses, to this end the DSM-5 Level of Personality Functioning Scale-Self Report (LPFS-SR) gives a comparable framework to assess personality functioning [18]. Along this line we hypothesized that ED and impulsivity can be potential mediators between childhood traumas and personality functioning.

To recapitulate, this study had three objectives: First, to compare factors of ED and impulsivity between the aADHD, BPD and healthy control groups, and to explore divergent inhibitory profiles. Second, to explore the association of childhood trauma and self-regulatory impairments at trait and behavioral levels. Third, to test the potential role of ED and impulsivity between childhood traumas and personality functioning. The rationale of our approach was to investigate ED and impulsivity both within and across diagnostic groups, and identify background factors and mediation effects.

## Methods

### Participants

Hundred patients receiving treatment for aADHD and 63 patients diagnosed with BPD (age range for both groups: 18–36 years) were enrolled in the study, and 100 healthy controls (HC) were matched according to age. Exclusion criteria included psychotic symptoms, intellectual developmental disorder, and visual or reading impairment. The socio-economic status (SES) was assessed by the Barratt Simplified Measure of Social Status [19]. The demographic and clinical characteristics of the three groups are presented in Table 1.

**Table 1:** Demographic and clinical characteristics of the aADHD, BPD, and HC groups

	aADHD (*N* = 100)	BPD (*N* = 63)	HC (*N* = 100)	F / χ²/H	*p*
Age (years, SD)	26.31 (4.71)	26.19 (4.51)	26.61 (4.59)	0.187	0.829
Sex (M/F) % ^*a*^	52.0/48.0	20.6/79.4	44.0/56.0	16.102	**< 0.001**
Level of education (E/S/H) % ^*b*^	4.0/51.0/45.0	7.9/61.9/30.2	3.0/49.0/48.0	6.309	**0.043**
SES (BSMSS, SD)^c^	50.07 (8.73)	43.63 (11.80)	48.55 (10.69)	17.103	**< 0.001**
Inattention/Memory problems (CAARS-A)	25.55 (6.35)	17.47 (5.80)	11.07 (5.94)	138.512	**< 0.001**
Hyperactivity/Restlessness (CAARS-B)	20.862 (6.74)	14.70 (5.96)	11.30 (5.86)	57.606	**< 0.001**
Impulsivity/Emotional lability CAARS-C)	18.98 (7.07)	18.57 (6.87)	9.61 (5.24)	61.921	**< 0.001**
Problems with self-concept (CAARS-D)	11.47 (4.06)	13.55 (3.524)	6.24 (3.75)	74.316	**< 0.001**
DSM-IV Inattentive Symptoms (CAARS-E)	19.01 (4.64)	12.50 (5.42)	6.59 (3.90)	179.782	**< 0.001**
DSM-IV Ha-Imp Symptoms (CAARS-F)	15.39 (5.39)	10.33 (4.34)	7.49 (4.64)	64.266	**< 0.001**
DSM-IV ADHD Symp. Total (CAARS-G)	34.40 (7.62)	22.83 (8.12)	14.07 (7.21)	173.292	**< 0.001**
ADHD index (CAARS-H)	23.20 (5.20)	19.32 (5.78)	10.35 (5.47)	138.954	**< 0.001**
Borderline Symptom Checklist – Behavior	16.73 (4.23)	22.52 (6.64)	14.65 (2.94)	49.385	**< 0.001**
Distractibility (PID-5) Distractibility	2.29 (0.53)	1.71 (0.77)	0.88 (0.58)	124.18	**< 0.001**
Impulsivity (PID-5) Impulsivity	1.34 (0.68)	1.48 (0.78)	0.63 (0.56)	36.012	**< 0.001**
Irresponsibility (PID-5)	1.36 (0.619)	1.12 (0.63)	0.65 (0.47)	37.854	**< 0.001**
*Comorbidity*					
Depression (%)	38.0	73.0	-		
Dysthymia (%)	5.0	22.2	-		
Suicidal risk: no/low/mid/high (%)	88.0/10.0/-/-	15.9/41.3/20.6/22.2	-		
(Hypo)mania (%)	-	3.2	-		
Anxiety (%)	27.0	76.2	-		
OCD (%)	1.0	7.9	-		
PTSD (%)	0.0	25.4	-		
Substance related (%)	12.0	22.2	-		
Anorexia (%)	0.0	7.9	-		
Bulimia (%)	0.0	11.1	-		
*Medication data*					
No medication (%)	43	20.6	84		
Methylphenidate (%)	49	-	-		
Atomoxetine (%)	1	-	-		
Bupropion (%)	2	-	-		
Other antidepressant (%)	4	55.6	-		
Antipsychotic (%)	-	30.2	-		
Mood stabilizer and Anticonvulsant (%)	-	30.2	-		
Anxiolytic (%)	-	31.7	-		
Other (%)	11	12.7	16		

^a^ M: Male, F = Female. ^b^ For completed education, E: elementary, S: secondary, H: higher education, ^c^ BSMSS: Barratt Simplified Measure of Socioeconomical Status, which accounts for an individual’s parent’s educational attainment and occupational prestige, OCD: Obsessive-compulsive disorder, PTSD: post-traumatic stress disorder

All patients were recruited at the Department of Psychiatry and Psychotherapy of Semmelweis University in Budapest, Hungary. A board-certified psychiatrist or clinical psychologist interviewed the patients in the BPD and aADHD groups using the MINI 5.0 [20, 21]. The Structured Clinical Interview for DSM-5 Personality Disorders (SCID-5-PD) was used to validate the clinical diagnosis and to detect potential comorbidities [22]. aADHD patients with comorbid BPD and BPD pateints with a history of present or past ADHD symptoms were not enrolled to avoid the presence of both disorders.

Control individuals without any psychiatric or substance use disorder history were recruited on social media platforms, at public events, and from hospital staff and their acquaintances. They were screened by the Derogatis Symptom Checklist (SCL-90) [23] and the Conners’ Adult ADHD Rating Scales (CAARS, 66-item version) [24]. To meet inclusion criteria for the HC group, the t-scores of the SCL-90 Global Severity Index, and the CAARS Inattention, Hyperactivity and Impulsivity domains had to be lower than 70. The CAARS and the Borderline Symptom Checklist’s behavioral items [25] were used to assess ADHD and borderline symptom severity in each group, respectively.

### Online questionnaires

All questionnaires were administered online. The Personality Inventory for DSM-5 (PID-5) [1, 26, 27] assessed traits of disinhibition (distractibility, impulsivity, irresponsibility). The 36-item Difficulties in Emotion Regulation Scale (DERS) scale yielded information on dimensions of nonacceptance, goal-directed behavior, impulsive behavior, emotion regulation, emotional awareness, and emotional clarity [28]. The 11th version of the Barratt Impulsivity Scale (BIS-11) [29, 30] was used to investigate attentional, motor, and non-planning impulsivity. With the LPFS-SR we assessed personality function components of Identity, Self-Direction, Empathy, and Intimacy, and the global dimension of personality dysfunction [18]. The Childhood Trauma Questionnaire-Short Form (CTQ-SF) was used to assess traumatization [2, 31].

### Neuropsychological tests measuring behavioral impulsivity and impulsive choice

To assess waiting and stopping impulsivity, selected tests of the Cambridge Neuropsychological Test Automated Battery (CANTAB) were used, namely the Reaction Time (RTI), and the Stop Signal Task (SST) [32]. Although the RTI is designed for measuring reaction time, it can also be used for measuring waiting impulsivity by analyzing the number and the probability of premature responses, when the subject cannot wait until the presentation of stimuli. The waiting impulsivity related outcome measures are the RTI Simple/Five choice Error Scores (premature responses), the total number of trials where the subject makes a response before the presentation of the target stimulus. In the SST the main outcome measure is Stop Signal Task Reaction Time (SSRT), which is an average time required for successful stopping; longer SSRT indicates greater difficulty in interrupting actions, reflecting on stopping impulsivity [33].

A computerized version of Rogers decision-making task was used to assess impulsive choice [34]. This task is designed to measure decision-making under uncertainty and delay aversion, as participants must make probabilistic judgments and make bets based on their confidence in their choice. The more detailed description of the test, and the process of the outlier detection process, has been described elsewhere [35].

### Statistical analysis

The SPSS version 28 [36] and JASP 0.19.1 [37] were used for all statistical analyses. Group differences were compared between BPD, aADHD and control groups using ANOVA and Bonferroni post hoc tests. Neuropsychological test results were compared by either ordinal/logistic regression, or univariate ANOVA. Delay aversion was analyzed by repeated measures ANOVA. All analyses were controlled for gender and SES. Predictors in the background of trait impulsivity factors were analyzed by linear regression, SES and gender were included in the model as covariates. The mediator model was created in SPSS, according to Process v4 by Andrew F. Hayes [38].

## Results

### Differences in demographic and clinical symptom characteristics

The three groups were balanced according to age, but a significant difference in gender ratios was observed due to the higher rate of women in the BPD group. There were also significant differences in SES and education levels, with lower levels of completed education and SES scores in the BPD group. Inattention/Memory problems (CAARS-A) and Hyperactivity/ Restlessness (CAARS-B) were the most characteristic to the aADHD group, but the BPD group showed significantly increased scores in these domains compared to the HC group (aADHD > BPD > HC). The DSM-IV ADHD scores (Inattention, CAARS-E; Hyperactivity-impulsivity, CAARS-F) showed the same pattern, indicating significant differences (aADHD > BPD > HC). Impulsivity/Emotional lability was equally characteristic for both patient groups (aADHD = BPD > HC). The BPD group scored the highest on the Problems of Self-concept scale (BPD > aADHD > HC). We found significant differences in every domain of the Borderline Symptom Checklist and PID-5, except for antagonism. All demographic and clinical results of the three groups are presented in Table 1.

### ED and trait impulsivity measures

**Fig. 1:**
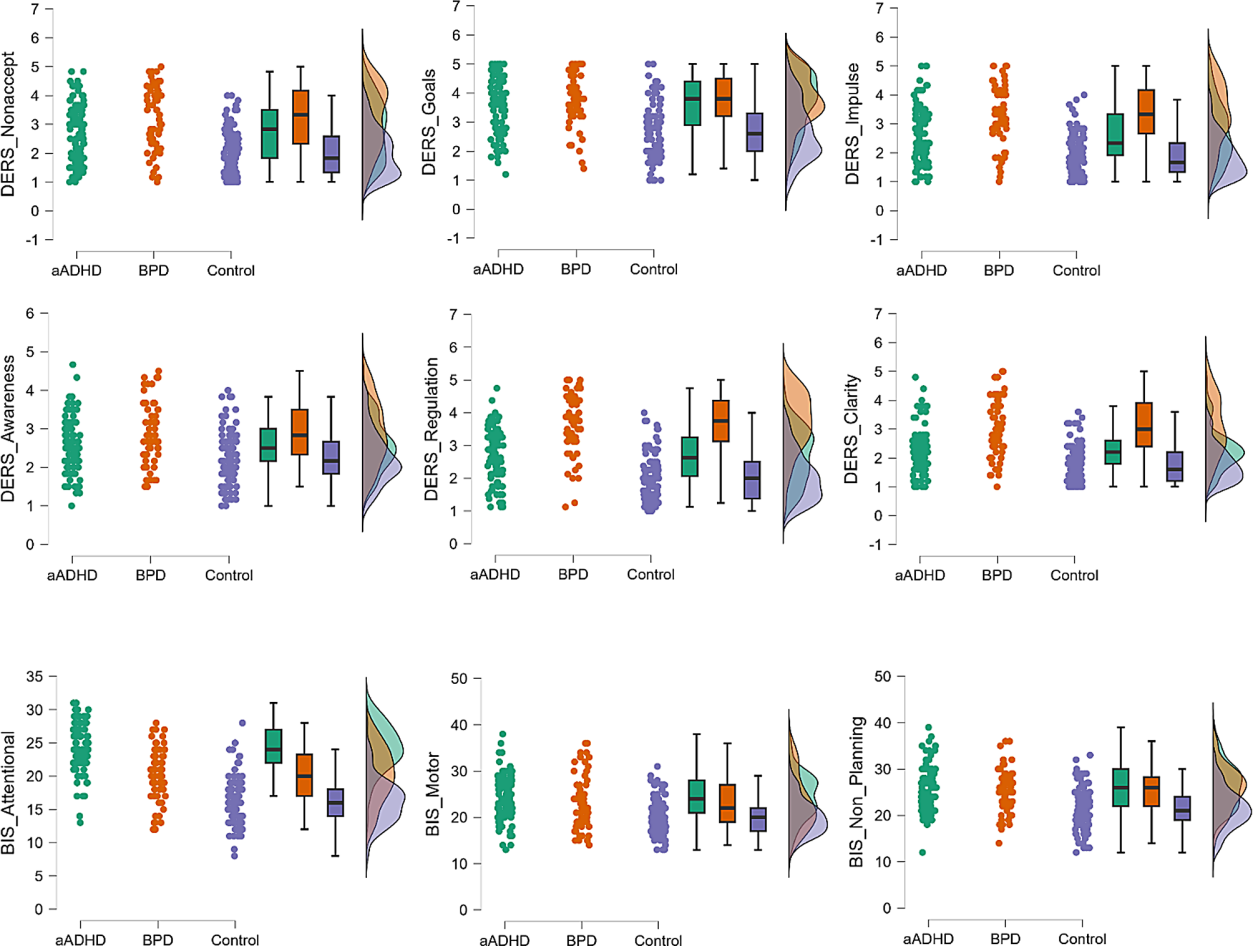
Difficulties in Emotion Regulation and trait impulsivity subscales in the aADHD, BPD, HC groups. DERS: Difficulties in Emotion Regulation Scale; BIS-11: Barratt Impulsiveness Scale 11th version

Total DERS and BIS-11 scores were significantly different in the three groups. The highest DERS total score was reported in BPD, followed by the aADHD group, and the lowest in the HC group (F(2,247) = 64.206; *p* < .001; part. η²=0.342). BIS-11 total score was significantly higher in aADHD compared to BPD and HC groups, while the difference between BPD and HC was also statistically significant (F(2,248) = 36.744; *p* < .001; part. η²=0.355). ED and impulsivity subscales are summarized in Fig. 1. The two patient groups were significantly different from the control group in each subscale, and they also differed from each other. In the ED subscales the BPD group reached higher scores than the aADHD group, accept for the nonacceptance, goals, and awareness subscales, which showed no significant difference between the two patient groups. Attentional impulsivity was more characteristic to aADHD, motor and non-planning impulsivity was similar in the two patient groups. All ED and impulsivity-related results of the three groups are presented in Table 2.

**Table 2:** Emotion dysregulation and impulsivity traits in the aADHD, BPD and HC groups

Variable	Group	*N*	M	S.D.	F (df1, df2)	*p*	partial η²	Contrasts	*p*
DERS	aADHD	94	2.746	1.027	29.266 (2, 247)	< 0.001	0.192	aADHD > HC	**< 0.001**
Nonacceptance	BPD	59	3.218	1.096				BPD > HC	**< 0.001**
	HC	99	2.003	0.800				aADHD = BPD	0.069
DERS	aADHD	94	3.655	0.963	31.082 (2, 247)	< 0.001	0.201	aADHD > HC	**< 0.001**
Goals	BPD	59	3.736	0.891				BPD > HC	**< 0.001**
	HC	99	2.687	0.978				aADHD = BPD	1.000
DERS	aADHD	94	2.612	1.005	42.503 (2, 247)	< 0.001	0.256	aADHD > HC	**< 0.001**
Impulse	BPD	59	3.314	1.069				BPD > HC	**< 0.001**
	HC	99	1.857	0.706				aADHD < BPD	**< 0.001**
DERS	aADHD	94	2.572	0.723	13.017 (2, 247)	< 0.001	0.095	aADHD > HC	**0.003**
Awareness	BPD	59	2.896	0.823				BPD > HC	**< 0.001**
	HC	99	2.227	0.689				aADHD = BPD	0.125
DERS	aADHD	94	2.646	0.845	62.808 (2, 247)	< 0.001	0.337	aADHD > HC	**< 0.001**
Regulation	BPD	59	3.659	0.936				BPD > HC	**< 0.001**
	HC	99	2.023	0.732				aADHD < BPD	**< 0.001**
DERS	aADHD	94	2.261	0.764	40.820 (2, 247)	< 0.001	0.248	aADHD > HC	**< 0.001**
Clarity	BPD	59	3.048	1.011				BPD > HC	**< 0.001**
	HC	99	1.778	0.639				aADHD < BPD	**< 0.001**
BIS Attention	aADHD	96	3.029	0.442	114.41 (2,248)	< 0.001	0.480	aADHD > HC	**< 0.001**
	BPD	60	2.527	0.514				BPD > HC	**< 0.001**
	HC	99	2.034	0.435				aADHD > BPD	**< 0.001**
BIS Motor	aADHD	96	2.224	0.441	22.071 (2, 248)	< 0.001	0.151	aADHD > HC	**< 0.001**
	BPD	60	2.106	0.526				BPD > HC	**0.003**
	HC	99	1.815	0.351				aADHD v BPD	0.092
BIS Nonplanning	aADHD	96	2.392	0.446	31.173 (2, 248)	< 0.001	0.201	aADHD > HC	**< 0.001**
	BPD	60	2.317	0.429				BPD > HC	**< 0.001**
	HC	99	1.937	0.390				aADHD = BPD	0.164

DERS: Difficulties in Emotion Regulation Scale, BIS: Barratt Impulsiveness Scale 11th version

### Neuropsychological measures of impulsivity and impulsive choice differences

Waiting impulsivity was measured by CANTAB Reaction Time paradigm’s premature responses. No significant difference was found between the three groups in the RTI simple error score variable. The five choice error score differed significantly between the aADHD and HC groups, while there was no significant difference between the BPD and HC groups in premature responses. In the stopping impulsivity measures, which represent the ability to stop ongoing actions, the aADHD group performed worse than the HC group. SSRT was significantly longer in the aADHD group than the HC group (Table 3). There was no significant difference between the BPD and HC groups.

**Table 3:** Comparison of impulsivity-related CANTAB variables in the aADHD, BPD, and HC groups

Variable name	aADHD	BPD	Control	Contrasts	Statistic value	*p*
RTI simple error score – premature responses ^1^	M = 0.43	M = 0.30	M = 0.28	aADHD v HC	b = 0.321	0.328
	S.D. = 0.728	S.D. = 0.638	S.D. = 0.533	BPD v HC	b = 0.177	0.664
				aADHD v BPD	b = 0.144	0.720
RTI five choice error score – premature responses ^2^ /dichotomized because of skewness/	Percentage of error: 14%	Percentage of error: 6.3%	Percentage of error: 5%	Main effect of group	χ² (2) = 4.836 R² = 1.9–4 0.2%	0.089
				aADHD > HC	OR = 3.012	**0.045**
				BPD v HC	OR = 1/0.685	0.599
				aADHD v BPD	OR = 2.064	0.255
Stop signal task reaction time (SSRT) ^3^ (measured in ms)	M = 242.230 S.D. = 42.880	M = 228.147 S.D. = 47.222	M = 219.172 S.D. = 37.778	Main effect of group	F(2,248) = 7.609	< 0.001
				aADHD > HC		**< 0.001**
				BPD v HC		> 0.999
				aADHD v BPD		0.081
SST Direction error – go trials^1^	M = 2.83	M = 1.54	M = 1.31	aADHD > HC		**0.019**
	S.D. = 4.568	S.D. = 2.657	S.D. = 2.509	BPD v HC		0.658
	Med: 1.00	Med: 1.00	Med. 0.50	aADHD v BPD		0.139
SST Direction error – stop trials^1^	M = 43.17	M = 42.21	M = 41.37	aADHD > HC		**< 0.001**
	S.D. = 4.725	S.D. = 4.080	S.D. = 3.762	BPD v HC		0.076
	Med: 43.00	Med: 43.00	Med. 41.00	aADHD v BPD		0.258

(1) Ordinal regression - comparison contains logistic regression slopes and significance values. Descriptive statistics contains Mean and standard deviation and median. (2) Logistic regression – we used Cox & Snell and Nagelkerke R² as effect sizes of main the effect of group. Comparison contains odds ratios (OR) and significance values. Descriptive statistics contains percentages. (3) Univariate ANOVA - comparison contains the main effect of group and Bonferroni Post hoc. Descriptive statistics contains Mean and standard deviation. All analyzes are controlled for age, gender, and SES

RTI: Reaction Time, SST = Stop Signal Task

**Fig. 2:**
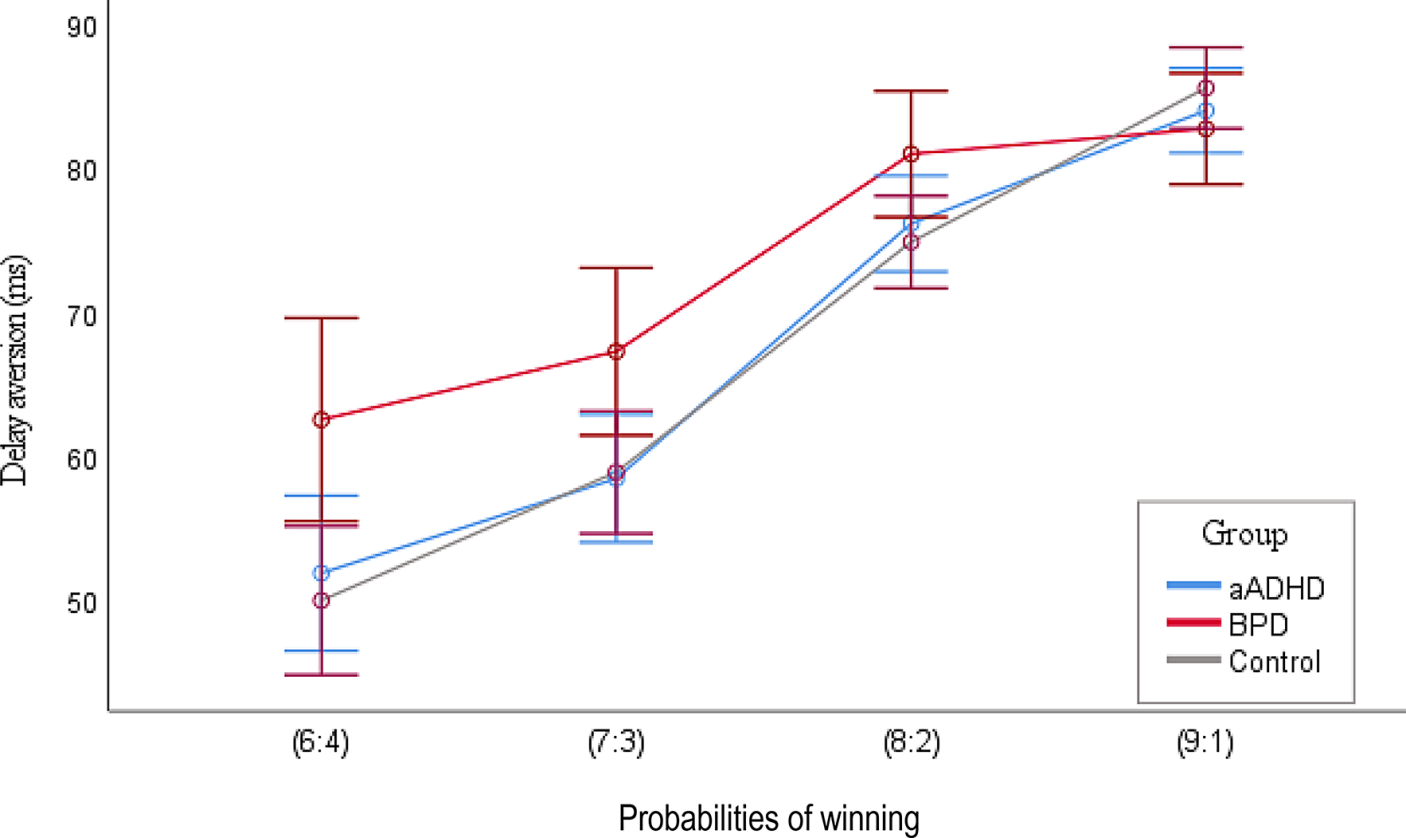
Delay aversion in aADHD, BPD, and HC groups as a function of probability of winning. On the X axis, the ratios mean the numbers of the red/blue boxes, therefore the probability of winning. Delay aversion reflects on the time subjects can wait in order to adjust the bet to the winning probability. The bets were presented in a descending order, in 5 steps

The decision making task has several outcome measures, of which only delay aversion was used for further analyses. Delay aversion represents the tendency of greater preference for smaller-immediate over larger-delayed rewards. The interaction of group and winning probability was significant (F(2,211) = 4.996; *p* = .008; part. η²=0.045), and subjects in the BPD group had the tendency of impatiently accepting larger bets earlier, despite the low probability of winning in comparison to the HC group (F(2,211) = 3.101; *p* = .047; part. η²=0.029), if the bets were presented in descending order (Fig. 2). Upon ascending conditions, there was no statistically significant difference between the three groups in delay aversion (F(2,211) = 0.344; *p* = .709; part. η²=0.003).

### The association of childhood traumatization with ED, trait and behavioral impulsivity

**Fig. 3:**
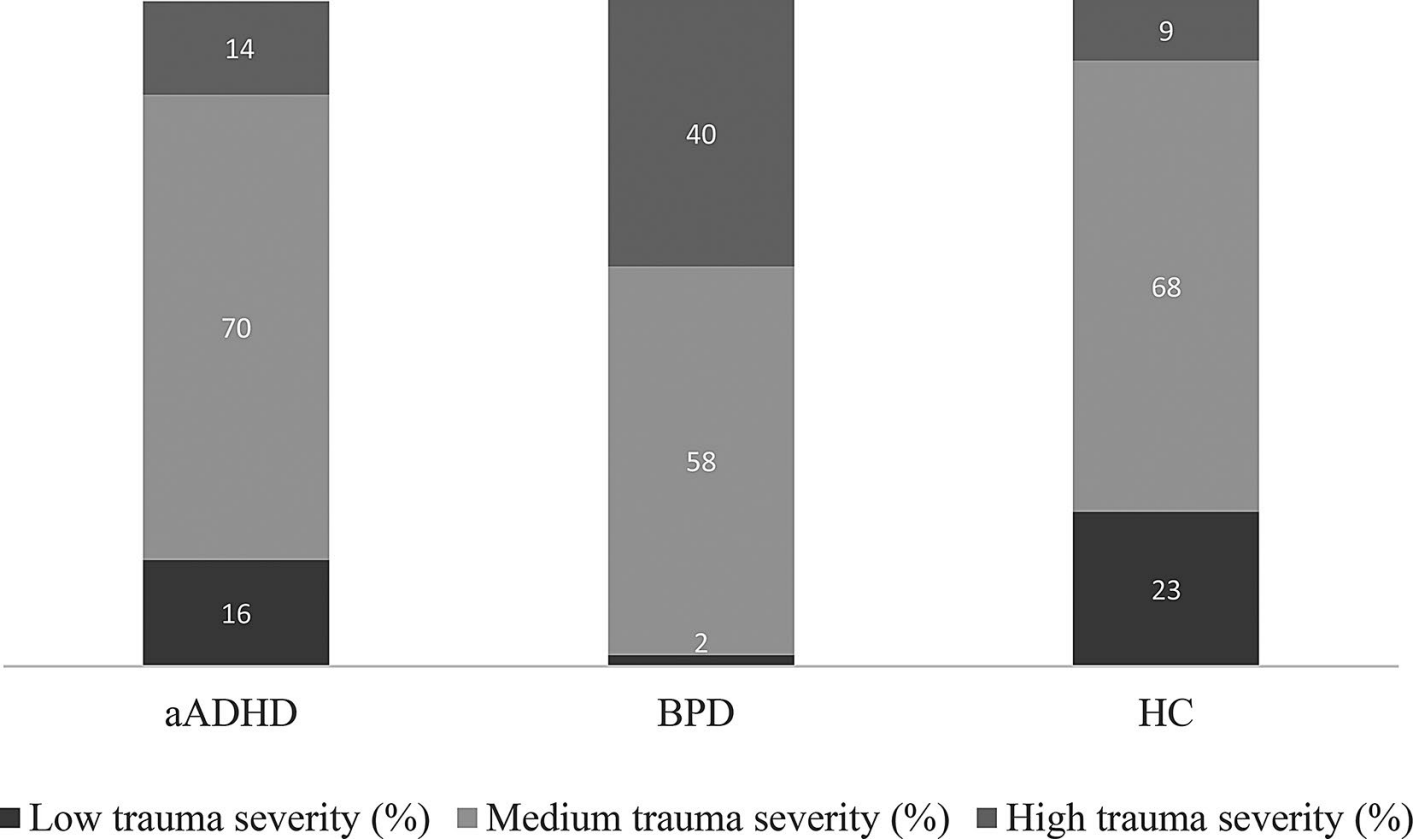
Distribution of traumatization categories in the aADHD, BPD, and HC groups. The grouping subjects into low, medium and high trauma severity groups was based on the CTQ mean total score, and +/- 1 SD. Low trauma severity: score under CTQ mean total score – 1 SD. Medium trauma severity: CTQ mean +/- 1 SD. High trauma severity: score above CTQ mean + 1 SD. CTQ: Childhood Trauma Questionnaire Short Form

Using the CTQ total score, a categorical trauma severity score was derived, grouping subjects into low, medium and high trauma severity groups, based on the mean CTQ total scores, and +/- 1 standard deviations as cut-off scores. As expected by clinical experience, the BPD group was characterized by the highest proportion of highly traumatized subjects. The distribution of traumatization levels was comparable between the aADHD and HC groups (Fig. 3).

**Fig. 4:**
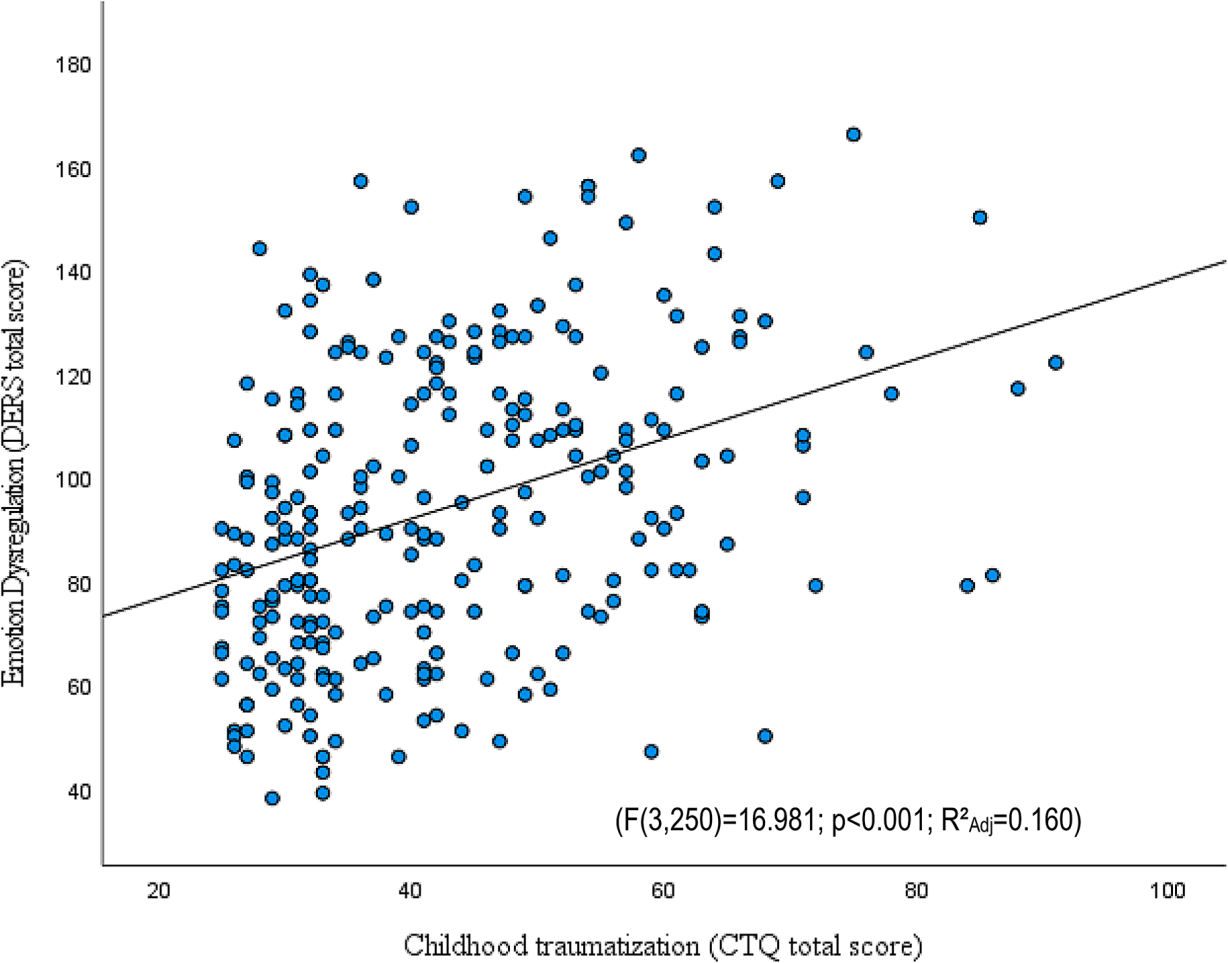
The effect of childhood traumatization on emotion dysregulation. CTQ: Childhood Trauma Questionnaire-Short Form; DERS: Difficulties in Emotion Regulation Scale

As a transdiagnostic dimensional approach to detect associations between childhood traumas, ED, and impulsive symptoms, we applied linear regression modeling. This offers a powerful method to assess psychopathology, beyond the structures of current diagnostic categories. According to our hypothesis, different factors of inhibitory capacity are associated with childhood traumatization to a different extent. Linear regression models were used for investigating to what extent gender, SES, and levels of traumatization predict trait impulsivity and ED.

**Table 4:** Sociodemographic and traumatization predictors of emotion dysregulation and trait impulsivity

DERS	B	SE	β	t	*p*	tolerance
Constant	66.281	11.636		5.696	< 0.001	
Gender	7.235	3.383	0.124	2.139	**0.033**	0.091
SES	− 0.288	0.160	− 0.106	-1.797	0.074	0.963
CTQ total	0.711	0.121	0.348	5.896	**< 0.001**	0.960
**BIS-11**	**B**	**SE**	**β**	**t**	**p**	**tolerance**
Constant	66.604	5.519		12.069	< 0.001	
Gender	− 0.859	1.617	− 0.032	− 0.531	0.596	0.092
SES	− 0.152	0.076	− 0.124	-2.002	.**046**	0.961
CTQ total	0.208	0.058	0.224	3.603	**< 0.001**	0.957

Linear regression models. Outcome variable: Emotion dysregulation (DERS): R^²^ = 0.170 R²_Adj_ = 0.160 F(3, 250) = 16.981 *p* < .001. Outcome variable: Trait impulsivity (BIS-11): R^²^ = 0.076 R²_Adj_ = 0.065 F(3, 250) = 6.840 *p* < .001. DERS: Difficulties in Emotion Regulation Scale, BIS: Barratt Impulsiveness Scale 11th version, SES: Socioeconomic status,, CTQ: Childhood Trauma Questionnaire – Short Form

The level of traumatization predicted ED and trait impulsivity. Gender predicted ED, but had no significant correlation with total impulsivity scores, while socioeconomic status demonstrated significant correlation with trait impulsivity, but not with ED. ED measured by DERS showed significant correlations with the CTQ total score (F(3,250) = 16.981; *p* < .001; R²_Adj_=0.160) (Fig. 4; Table 4, and Supplementary Table 1), while total impulsivity scores measured by the BIS-11 were also correlated with the CTQ total score (F(3,250) = 6.840; *p* < .001; R²_Adj_=0.065) (Table 4 and Supplementary Table 2). Waiting and stopping impulsivity showed no differences as a function of the level of traumatization. Delay aversion level among uncertain conditions differed significantly according to the level of traumatization, namely, those who were the most traumatized, had the highest delay aversion scores (F(2,169) = 3.192; *p* = .044; part. η²=0.036).

**Fig. 5:**
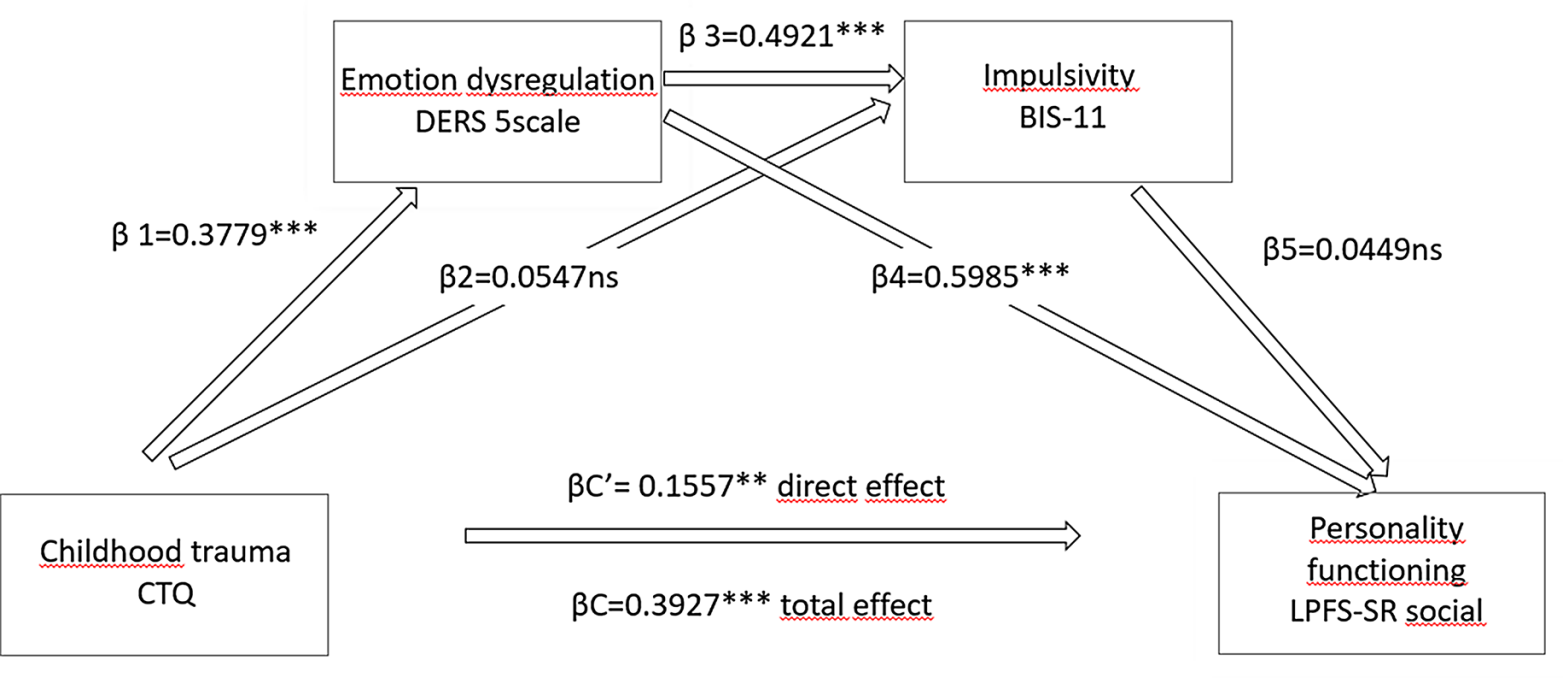
Emotion dysregulation and impulsivity mediate between childhood traumatization and personality functioning. CTQ: Childhood Trauma Questionnaire Short Form; BIS: Barratt Impulsivity Scale 11th version; DERS: Difficulties in Emotion Regulation Scale; LPFS - SR social: Level of Personality Functioning Scale - Self Report form social domain

### ED and impulsivity as mediators between childhood traumas and personality functioning

To assess the role of ED and impulsivity in the background of personality functioning, a mediator model was implemented. Childhood traumatization as predictor variable, ED and impulsivity as mediator variables, and personality functioning as outcome variable were predefined. We excluded the Self domain of LPFS from the analysis, because of the overlap in self-regulatory items with DERS and BIS-11. We created a 5-scale version from the DERS, while the “impulse” scale of DERS refers to impulsive behaviour. Each variable was transformed to z-scores and corrected for sign in downstream analyses. Standardized coefficients demontrated that traumatization had a significant total and direct effect on personality functioning. Among the indirect pathways, only those remained significant, which contained ED as a mediator. The model also showed that the detected associations between trait impulsivity and childhood traumatization were mediated by ED (Fig. 5).

## Discussion

ED, impulsivity, and according to the self-reported results of our study, even inattention, are overlapping symptoms of aADHD and BPD, however, disorder-specific characteristics of these constructs have not been clearly defined yet. In this study we assessed the inhibitory profile of both patient groups avoiding the bias effect of aADHD-BPD comorbid cases.

From a clinical point of view, DSM-5 diagnostic criteria show significant differences in the definition of impulsivity for these disorders. The DERS and BIS-11, measures of self-reported inhibitory capacity showed marked differences between the aADHD, BPD, and HC groups. The DERS total score was the highest in the BPD group, and the lowest in the HC group. The aADHD group demonstrated significant differences compared to both the BPD and HC groups. In each DERS subscale the BPD group reached the highest scores nominally, however the impulse (controlling impulsive behavior when distressed), regulation (limited access to effective emotion regulation strategies) and clarity (lack of emotional clarity) scales showed significant difference between the two patient groups. The impulse subscale of DERS has the most overlap with the “negative urgency concept”, affective instability driven impulsivity. In the study of Krause-Utz et al. not only the BPD, but also the clinical control group expressed increased negative urgency [16]. Linhartová et al. found that the only significant trait impulsivity difference between BPD and aADHD patients was negative urgency, with higher scores in BPD patients [7]. The regulation and clarity subscale of DERS refers to the capability of recognizing emotions and the lack of adaptive regulation strategies. The elevated DERS scores found in BPD relative to aADHD are in line with previous findings of Rüfenacht et al., who found that aADHD patients have a better control over their emotions with higher use of adaptive cognitive strategies and lesser use of non-adaptive strategies than BPD patients [14].

The BIS-11 impulsivity total scores were the most characteristic for the aADHD group in our study, the BPD group demonstrated medium level scores, differing significantly from aADHD and HC groups as well, similarly as reported by Lepouriel et al. [8]. As predicted, the BIS-11 attentional scores were significantly higher in aADHD compared to BPD and HC groups. In summary, the self-regulatory profile, measured by self-reported questionnaires differs in these groups. As expected, ED was more prominent in BPD, while impulsivity was more characteristic to aADHD.

Using the Conners’ Adult ADHD rating scale the aADHD group scored the highest in each domain, except for the Problems with self concept scale. Nevertheless, the BPD group also had significantly increased scores in each ADHD domain when compared to the HC group. The CAARS measures Impulsivity and Emotional lability on one subscale (CAARS-C), therefore the disorder specific differences between theses two features, which we were able to detect with the DERS and the BIS-11 separately were not observable here.

A potential explanation of the ADHD-like characteristics found in BPD by self-report questionnaires can be trauma-related. Traumatization can make children feel agitated, troubled, nervous, or alert and restless. These behaviors can be mistaken for hyperactivity. Symptoms that seem like inattention or concentration problems in children who experience trauma can be symptoms of dissociation or the result of avoidance of trauma reminders [39]. A similar mechanism is possible in adults living with BPD.

Waiting impulsivity was measured with the RTI subtest of CANTAB. The aADHD group showed elevated level of waiting impulsivity, while the BPD group did not differ from healthy controls in premature responses of RTI. In former studies, individuals with BPD have been found to exhibit heterogeneous results in neuropsychological tests, because of the high rate of comorbidity, including affective disorders, substance use, and ADHD [40]. Recently, several studies have found intact waiting and stopping impulsivity in BPD under emotionally neutral circumstances [9, 41, 42]. In our study, stopping impulsivity was increased in aADHD, but not in BPD, which corresponds to previous studies [10, 43]. Delay aversion measured by the Rogers decision making task was detected only in the BPD group. The difference was significant during descending conditions, while the probability of winning was very low. In other words, despite the uncertain conditions the BPD patients took earlier, therefore larger bets than the HC group. The aADHD group was similar to the HC group and had a similar delay aversion profile. Our results support previous findings, which found delay aversion relevant to BPD only under specific conditions [41–43]. Taken together, our results corroborate the multifaceted and disease-specific nature of impulsive behavior [44].

Traumatic events in childhood, especially those that influence emotional maturation, are considered as a predisposing factor for the later development of ED and impulsivity (reviewed by Calvo et al.) [45]. The results of our transdiagnostic linear regression analyses support these findings, with trauma scores predicting both ED and impulsivity traits. In the case of ED, gender was also a significant predictor, while for impulsivity, SES was a significant predictor.

We found no associations between waiting and stopping impulsivity and the level of traumatization, which might be a consequence of the sex-dependent nature of maltreatment-related reorganization of the brain inhibitory control network resulting in poorer response inhibition among males [46]. In our sample females were overrepresented to males, which might be the reason, why stopping impulsivity seems to be intact in different BPD samples throughout studies [9, 10]. Delay aversion level among uncertain conditions differed significantly according to the level of trauma. Those who were most traumatized, had the highest delay aversion scores, regardless of their diagnosis. This association gives a potential insight to coping with a chaotic, traumatizing milieu, which was characteristic to the BPD group to a greater extent. Where the future is not predictable, the short term gains becomes more important and therefore it can be considered as a relevant coping strategy.

Several studies have suggested that traumatic childhood experiences are associated with personality disorders, depression, anxiety, addictions, suicidal behavior, obesity [47–52], but according to our best knowledge, there has not been studies published about the mediating effect of ED or impulsivity between traumatization an DSM-5 personality functioning. Our aim was to assess the role of traumatization across diagnostic categories, and find potential mediators in a transdiagnostic analysis. In our sample, childhood traumatization had a significant total and direct effect on adult personality functioning (LPFS-SR, social functioning), but among the indirect pathways only those were significant which contained ED as a mediator. ED seems to mediate the effects of traumatization on impulsivity as well. However, it is crucial to acknowledge that the correlational design applied does not allow causal conclusions to be drawn between childhood traumatization, ED, impulsivity, and personality functioning. Therefore, it is essential for future research to investigate theses phenomena in prospective, well-designed studies.

Limitations of our study need to be acknowledged. We cannot report about aADHD - BPD comorbid cases, because they were not involved in our research study. The aADHD and BPD patients recruited to the study already underwent clinical screening previously as their standard diagnostic procedure, therefore the patients with a comorbid diagnosis of BPD or ADHD were not approached, so we cannot report the exact number of these cases. According to the MINI 5.0 interview, the prevalence of comorbidies were higher at the BPD group than in the ADHD group, which has a potential impact on the impulsivity measures. The sex ratio was different in the aADHD and BPD group, but we included sex as cofactor in each analysis.

Another limitation regarding the assessment tools is that three of the questionnaires haven’t been validated in Hungarian language. Therefore, the results of SCID-5-PD, the Conners’ Adult ADHD Rating Scales 66 item version (CAARS) and the DSM-5 Level of Personality Functioning Scale – Self Report form (LPFS-SR) should be interpreted with caution. In addition, results of self-reported scales are subjective, therefore can be distorted and there are more suitable self–reported scales for measuring impulsivity, i.e. the UPPS-P Impulsive Behavior Scale, which measures negative urgency, a factor which seems to be the best in distinguishing BPD from aADHD. The concept of negative urgency combines affective instability and impulsivity, and the importance of this combination in BPD has been emphasized in previous studies [7].

## Conclusions

In conclusion, assessing the inhibitory profile in aADHD and BPD resulted in a deeper understanding of the charcteristics of ED and impulsivity in these two disorders. The clinically observed symptom overlap covers relevant disorder specific characteristics. Taking into consideration ED and impulsivity profiles, and traumatization levels during differential diagnostics can serve as a basis for therapeutic approaches.

According to these results, in clinical settings ED and impulsivity are useful variables for differentiating aADHD from BPD, by evaluation with questionnaires and neuropsycholgical tests. Self-reported symptoms of inattention, restlessness, and impulsivity in BPD can be either signs of potential ADHD comorbidity, or childhood trauma related symptoms, or both. The evaluation of the level of traumatization is essential in differential diagnostics and in treatment planning. Finally, targeting ED by psychoeducation, pharmacological treatment or psychotherapy may have a valuable impact on personality functioning. Future research should explore longitudinal associations and the effect of clinical intervention in these patient groups.

## Electronic supplementary material

Below is the link to the electronic supplementary material.

Supplementary Material 1

## Data Availability

The datasets used and analysed during the current study are available from the corresponding author on reasonable request.

## Abbreviations

ADHD: Attention-Deficit/Hyperactivity Disorder
aADHD: adult Attention-Deficit/Hyperactivity DisorderANOVA: analysis of variance
BIS-11: Barratt Impulsiveness Scale 11th version
BPD: Borderline Personality Disorder
CAARS: Conners’ Adult ADHD Rating Scales
CANTAB: Cambridge Neuropsychological Test Automated Battery
CTQ-SF: Childhood Trauma Questionnaire-Short Form
DERS: Difficulties in Emotion Regulation Scale
DSM-5: Diagnostic and Statistical Manual of Mental Disorders 5th Edition
ED: Emotion Dysregulation
HC: Healthy Control
LPFS-SR: Level of Personality Functioning Scale -Self Report form
MINI: Mini-International Neuropsychiatric Interview
PID-5: Personality Inventory for DSM-5
RTI: Reaction Time
SCID-5-PD: Structured Clinical Interview for DSM-5 Personality Disorders
SCL-90: Derogatis Symptom Checklist-90
SES: Socioeconomic status
SPSS: Statistical Package for Social Sciences
SSRT: Stop Signal task Reaction Time
SST: Stop Signal Task
