# Supplementary material for: Emotion dysregulation and impulsivity as overlapping symptoms in adult Attention-Deficit/Hyperactivity Disorder and Borderline Personality Disorder: severity profiles and associations with childhood traumatization and personality functioning
Source: Ann Gen Psychiatry. 2025 Jan 13;24:3. doi: 10.1186/s12991-024-00540-y (PMC11730133; doi:10.1186/s12991-024-00540-y)
Supplement: Supplementary file 1 — Supplementary Material 1 [file 12991_2024_540_MOESM1_ESM.docx]

**Supplementary material to Emotion dysregulation and impulsivity as overlapping symptoms in adult Attention-Deficit/Hyperactivity Disorder and Borderline Personality Disorder: Severity profiles and associations with childhood traumatization and personality functioning by Kenézlői et al.**

**Supplementary Table 1.** Descriptive statistics of the regression model.

Outcome variable: DERS; Predictors: Gender, SES, CTQ

After carrying out the outlier detection, the descriptive statistics of the variables used in the regression variables are in Supplementary Table 1**.**

|  | | | | | | | |
| --- | --- | --- | --- | --- | --- | --- | --- |
|  | | DERS | | SES | | CTQ | |
| Valid |  | 252 |  | 252 |  | 252 |  |
| Missing |  | 0 |  | 0 |  | 0 |  |
| Mean |  | 94.214 |  | 49.164 |  | 43.04 |  |
| Std. Deviation |  | 28.717 |  | 10.560 |  | 14.053 |  |

**Supplementary Figure 1.** Collinearity diagnostics of the regression model.


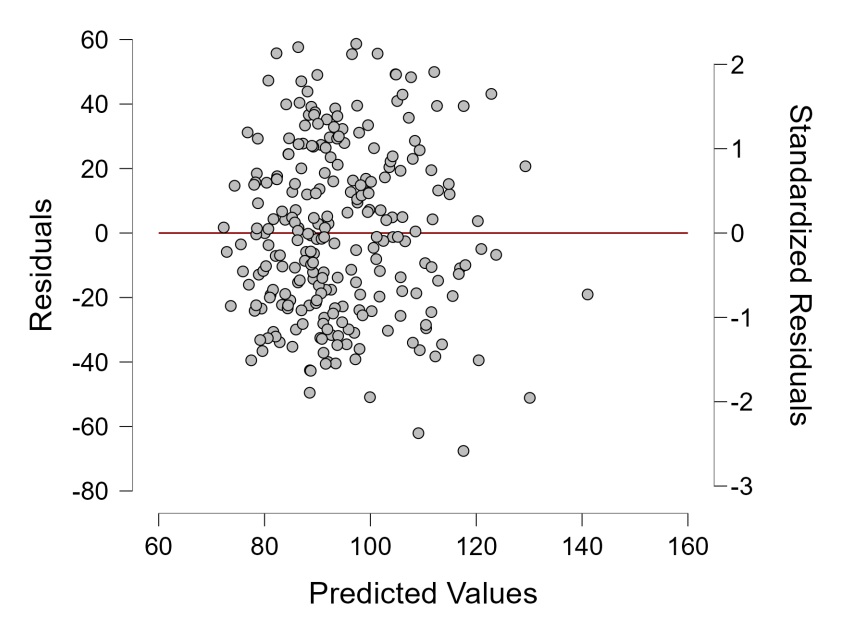

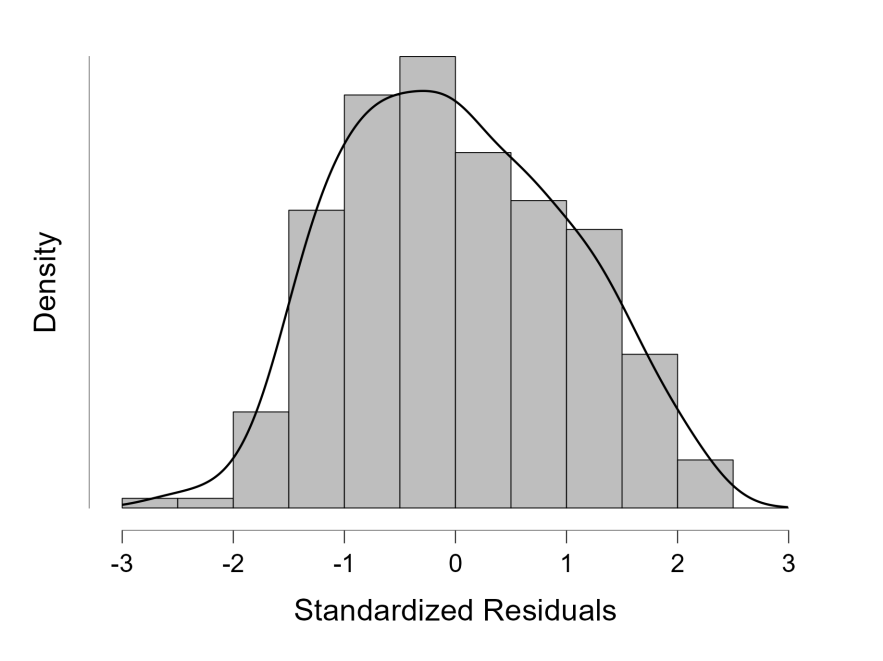


According to collinearity diagnostics, there is no collinearity and multicollinearity among the predictors. The correlation between the predictors: -0.194. The tolerance measures for gender = 99.1% SES=96.3%, CTQ=96.0%. The independence of the predictors makes it possible to detect their prediction value clearly in the regression. Based on the point diagram of the standardized predicted values and standardized residuals meet the requirements of homoscedasticity and linearity. The histogram of the standardized residuals shows normal distribution.

**Supplementary Table 2.** Descriptive statistics of the regression model.

**Outcome variable: BIS-11;** Predictors: Gender, SES, CTQ

After carrying out the outlier detection, the descriptive statistics of the variables used in the regression variables are in Supplementary Table 2.

|  | | | | | | | |
| --- | --- | --- | --- | --- | --- | --- | --- |
|  | | BIS-11 | | SES | | CTQ | |
| Valid |  | 254 |  | 254 |  | 254 |  |
| Missing |  | 0 |  | 0 |  | 0 |  |
| Mean |  | 66.764 |  | 49.025 |  | 43.063 |  |
| Std. Deviation |  | 13.091 |  | 10.682 |  | 14.063 |  |

**Supplementary Figure 2.** Collinearity diagnostics of the regression model.

###
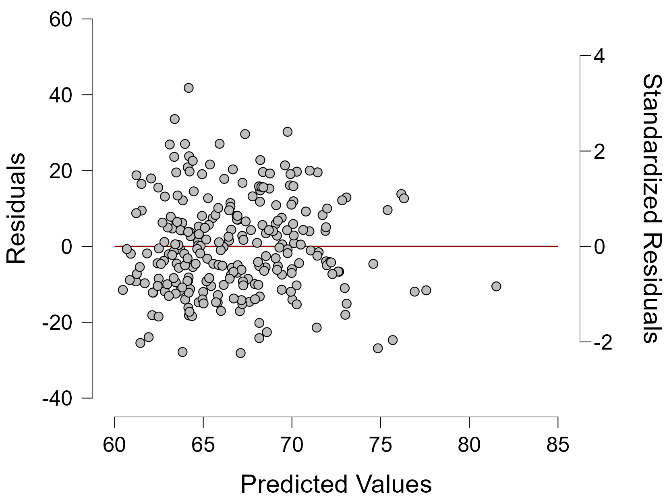

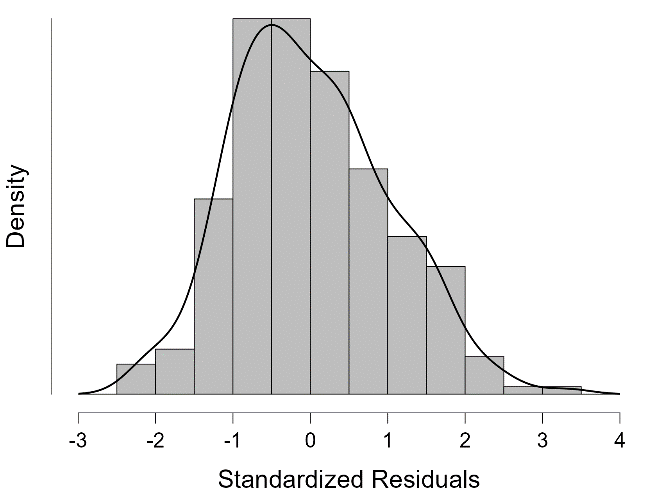


According to collinearity diagnostics, there is no collinearity and multicollinearity among the predictors. The correlation between the predictors: -0.194. The tolerance measures for gender = 99.2% SES=96.1%, CTQ=95.7%. The independence of the predictors makes it possible to detect their prediction value clearly in the regression. Based on the point diagram of the standardized predicted values and standardized residuals meet the requirements of homoscedasticity and linearity. The histogram of the standardized residuals shows normal distribution.
